# Investigation of the Association Between History of Learning Disabilities and Primary Progressive Aphasia in Brazilian Portuguese Speakers

**DOI:** 10.3389/fneur.2022.703729

**Published:** 2022-02-28

**Authors:** Talita Gallas dos Reis, Thais Helena Machado, Paulo Caramelli, Francisco Scornavacca, Liana Lisboa Fernandez, Bárbara Costa Beber

**Affiliations:** ^1^Departamento de Fonoaudiologia, Programa de Pós-Graduação em Ciências da Reabilitação, Universidade Federal de Ciências da Saúde de Porto Alegre - UFCSPA, Porto Alegre, Brazil; ^2^Departamento de Fonoaudiologia, Universidade Federal de Minas Gerais - UFMG, Belo Horizonte, Brazil; ^3^Departamento de Clínica Médica-Neurologia, Universidade Federal de Minas Gerais–UFMG, Belo Horizonte, Brazil; ^4^Departamento de Pediatria, Programa de Pós-Graduação em Pediatria: Atenção à Saúde da Criança e do Adolescente, Universidade Federal de Ciência da Saúde de Porto Alegre - UFCSPA, Porto Alegre, Brazil; ^5^Departamento de Ciências Básicas da Saúde, Universidade Federal das Ciências da Saúde de Porto Alegre–UFCSPA, Porto Alegre, Brazil

**Keywords:** primary progressive aphasia, Alzheimer's disease, learning disabilities, dyslexia, differential diagnosis

## Abstract

Primary Progressive Aphasia (PPA) is a neurological syndrome characterized by impaired language due to neurodegeneration. It is subdivided into three variants: semantic, agrammatic or nonfluent, and logopenic. Pieces of evidence have suggested that learning disabilities in childhood, such as dyslexia, might be susceptibility factors in the occurrence of PPA in adulthood. The objective of this study was to verify the existence of the relationship between PPA and the history of learning disabilities of patients and their children, compared to a control group of individuals with Alzheimer's disease (AD). A questionnaire was applied to investigate the presence of indicators of learning disabilities and difficulties in individuals with PPA and AD and their children. Twenty subjects with PPA and 16 with AD participated in the study. Our findings are presented and discussed in light of the current scientific evidence and the social, educational, and economic Brazilian scenario. Despite the challenges of doing research with individuals with PPA in Brazil, we present the first evidence about the investigation of association between the history of learning disabilities and difficulties and PPA in native Brazilian Portuguese speakers.

## Introduction

Primary Progressive Aphasia (PPA) is a neurodegenerative syndrome characterized by language impairment due to the degeneration in the frontal and temporal regions, mainly in the left hemisphere (1–4). PPA is confirmed in the presence of the insidious onset of language impairment, presence of a neurodegenerative process, and emergence of language complaints without significant impairments in other cognitive domains (3, 5). PPA is subdivided into three variants, which have specific diagnostic criteria and characterization (1, 2, 4, 6). The semantic variant (svPPA) presents with fluent spontaneous speech, occurrence of anomia episodes, and difficulty in understanding single words. It is caused by atrophy in the temporal pole of the left and/or right hemispheres. The nonfluent/agrammatic variant (nfvPPA) is characterized by apraxia of speech, agrammatism, and interrupted and disfluent speech. It presents atrophy in the frontal or insular brain regions of the left hemisphere. The logopenic variant (lvPPV), is characterized by difficulty in repeating sentences and in finding words, with occurrence of phonological errors in speech. In this variant, the neurodegeneration predominates in the left temporoparietal junction.

The growing research in the field of PPA has shown the possible existence of some susceptibility factors to the development of PPA (7), including learning disorders in childhood and suggesting that dyslexia might be a risk factor in the occurrence of the lvPPA. In addition, other secondary factors, such as traumas (traumatic brain injuries or tumors), history of vasectomy, and neurodegenerative diseases in the family, were also discussed in the literature as possible risk factors in the development of PPA (7, 8). Regarding the learning disabilities, there is a possibility that some genes responsible for the development of dyslexia may be linked to PPA (7); and that dyslexia may remain compensated for most of life manifesting as PPA in adulthood through a selective vulnerability of the language cortex to neurodegeneration (7, 9). It is known that learning disabilities such as dyslexia present neuroimaging findings with impairments in the brain areas responsible for the oral and written language abilities, mainly in the left hemisphere (10). This may justify the vulnerability of these brain areas, which become more susceptible to the development of neurodegenerative diseases, such as PPA.

The occurrence of learning disabilities among people with PPA and their first-degree relatives is significantly higher than among other neurodegenerative diseases, such as Alzheimer's disease (AD) or the behavioral variant of frontotemporal dementia (FTD) (11). It is important to understand if this finding would be found in other populations, especially in non-English speakers since PPA is a language-based disorder. In the case of Brazil, the early detection of PPA is difficult as well as the accurate diagnosis due to our social, economic, and educational scenario (12). As a consequence, it is difficult to recruit this population for research, and there is a scarcity of studies characterizing the Brazilian population with PPA (13). Given this context, and in order to collaborate with the characterization of the PPA Brazilian Portuguese speakers, the aim of this research was to investigate the relationship between PPA and the history of learning difficulties in the childhood of patients and their children in comparison to a control group with AD.

## Methods

### Participants

This is a quantitative, descriptive, and cross-sectional study. The sample was collected for convenience. The participants in this study were proceeding from the Neurology Outpatient Clinic of the Irmandade Santa Casa de Misericórdia de Porto Alegre (ISCMPA), and the Outpatient Clinic for Cognitive Neurology and Behavior at the Hospital das Clínicas da Universidade Federal de Minas Gerais (HC-UFMG).

The patients diagnosed with PPA (1) and AD (14) were included in the study, following the consensus of current diagnostic criteria, and who had a family member or a caregiver who knew the patient well enough to answer the questionnaire. The participants or their responsible caregivers consented to participate in the study by signing the informed consent form (ICF). Those who did not agree to participate in the study and those who did not have a family member or a responsible caregiver to answer the questionnaire were excluded from the study.

The neurological diagnosis of all the participants was made by a neurologist expert in cognitive disorders. For the diagnosis process, the neurologist considered information from interview with a patient and a caregiver; physical examination; neuropsychological and speech and language assessment (this is only for patients in suspicion of PPA); blood tests; and neuroimaging tests (magnetic resonance imaging-MRI). Some participants carried out cerebrospinal fluid examination with dosage of biomarkers of AD and functional neuroimaging tests (FDG-PET or SPECT). Our sample consisted of patients from both the public and private health systems. The Brazilian public health system does not cover the costs of the cerebrospinal fluid examination for AD biomarkers or functional neuroimaging, so such tests were only performed by the patients who could afford to pay for these tests privately or had health insurance to cover their costs. A few patients from the public universities had results of AD biomarkers in the cerebrospinal fluid as part of other research protocols.

This study was conducted in line with local ethical standards and was approved by the ISCMPA ethics committee (#3.117.790), and also by the HC-UFMG ethics committee (#2.018.855).

### Procedures

The researchers reviewed the medical records of the patients from the outpatient clinics involved in this study to select potential participants according to the criteria described above. Afterwards, the participants were invited to participate in the study and those who agreed were submitted to the collection of specific data for this study.

Data collection was performed through a structured questionnaire about history of learning disabilities/difficulties prepared by the researchers. To construct the questionnaire, the researchers were based on their experience and literature that suggest that there are barriers in the population's access to the detection and diagnosis of learning disorders, especially in the older generations, who are a large part of the participants in this study. Based on this, the researchers thought to include questions that could be suggestive of learning difficulties since the diagnosis was probably not possible.

First, the questionnaire was applied as a pilot in 5 dementia caregivers to verify if the questions were understandable. The authors made the corrections needed, and then the questionnaire was applied as an interview with a family member or a caregiver who had the best knowledge about the patient. The patients were asked to accompany the interview, but also that a caregiver was present together to transmit the information to the interviewer due to the participants' communication difficulties. The questionnaire was applied in person, or by telephone contact previously scheduled. The participants were informed about the study procedures, and then read and signed the ICF, and finally answered the interview. When the contact was made by telephone call, the ICF was read and agreed through an online document.

The questionnaire consisted of three blocks of questions, most of which were closed questions: 1. Personal and sociodemographic information; 2. Clinical data of the disease-PPA and AD; and 3. History of learning disabilities or difficulties. As it was, to our knowledge, that, in Brazil, at the time when the participants attended school, the investigation and the diagnosis of learning disorders were probably infrequent and very difficult, the questions of the questionnaire were not only about the occurrence of diagnosis of those disabilities but also questions that could infer that those people had learning difficulties (See the questionnaire in Appendix 1). The options of answers in the third section were categorized as “yes”, “no”, and “I do not know”.

The questionnaire was administered by one of the researchers, who is a speech and language pathologist with expertise in dementia and a Brazilian-Portuguese native speaker. This examiner was not involved in any part of the diagnostic process of our participants since they were already diagnosed when they were selected to participate in this study. The questionnaire took about 15 min to be administered.

### Data Analysis

Pearson's Chi-Square test and Fisher's exact test were used to investigate the existence of an association between history of indicatives of learning disabilities/difficulties and the participant's diagnosis. A significance level of 5% was adopted.

## Results

Twenty subjects with PPA participated in the study group, 8 with svPPA, 7 with nfvPPA, 3 with lvPPA, and 2 with non-classifiable PPA (*n* = 2). The control group was composed of 16 individuals with AD. The participants' descriptive data are shown in Table 1.

**Table 1:** Sociodemographic characteristics of the participants.

	**PPA (total)**	**Semantic PPA**	**Logopenic PPA**	**Nonfluent PPA**	**Non-classifiable PPA**	**AD**
	**(***N*** = 20)**	**(***N*** = 8)**	**(***N*** = 3)**	**(***N*** = 7)**	**(***N*** = 2)**	**(***N*** = 16)**
Sex (F)–*N* (%)	10 (50.0)	2 (25.0)	2 (66.7)	4 (57.1)	2 (100)	10 (62.5)
Age-mean (SD±)	68.1 (7.7)	65.0 (8.5)	67.0 (9.6)	72.4 (5.9)	67.0 (2.8)	79.9 (9.0)
Age of first symptoms–mean (SD±)	63.0 (8.6)	59.7 (3.1)	64.0 (6.2)	66.4 (3.3)	63.5 (6.2)	68.8 (8.4)
Educational level–average (SD±)	13.5 (4.3)	13.9 (3.6)	13.3 (4.6)	13.3 (5.0)	13.0 (8.5)	5.2 (4.0)
Hand dominance (right-handed)–*N* (%)	20 (100)	8 (100)	3 (100)	7 (100)	2 (100)	16 (100)
Race–*N* (%)
White	18 (90.0)	7 (87.5)	3 (100)	6 (85.7)	2 (100)	10 (62.5)
Mixed	2 (10.0)	1 (12.5)	0 (0)	1 (14.3)	0 (0)	3 (18.8)
Black	0 (0)	0 (0)	0 (0)	0 (0)	0 (0)	2 (12.5)
Indigenous	0 (0)	0 (0)	0 (0)	0 (0)	0 (0)	1 (6.3)

*PPA, primary progressive aphasia; AD, Alzheimer's disease; F, female; SD, standard deviation*.

Statistical comparisons on demographic variables were applied between AD and PPA groups. There was no significant difference for sex (*p* = 0.51) and race (*p* = 0.16), but the PPA group was significantly younger (*p* < 0.01) and with more years of education (*p* < 0.01).

**Figure 1:**
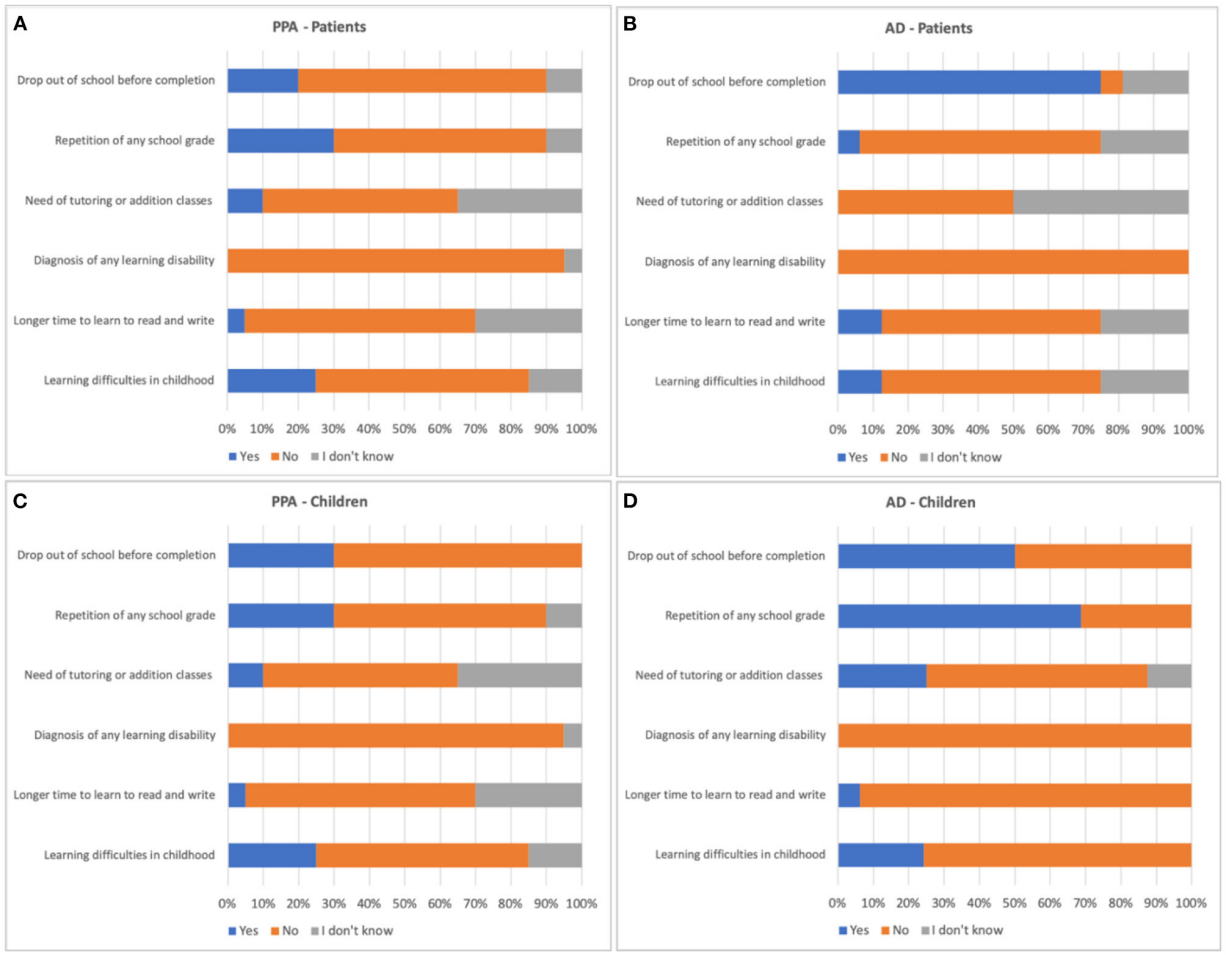
Description of the percentage of responses in the questionnaire of learning difficulties history of the AD and PPA groups. AD, Alzheimer's disease; PPA, primary progressive aphasia. **(A)** responses of PPA patients; **(B)** responses of AD patients; **(C)** responses of PPA children; **(D)** responses of AD children.

**Figure 2:**
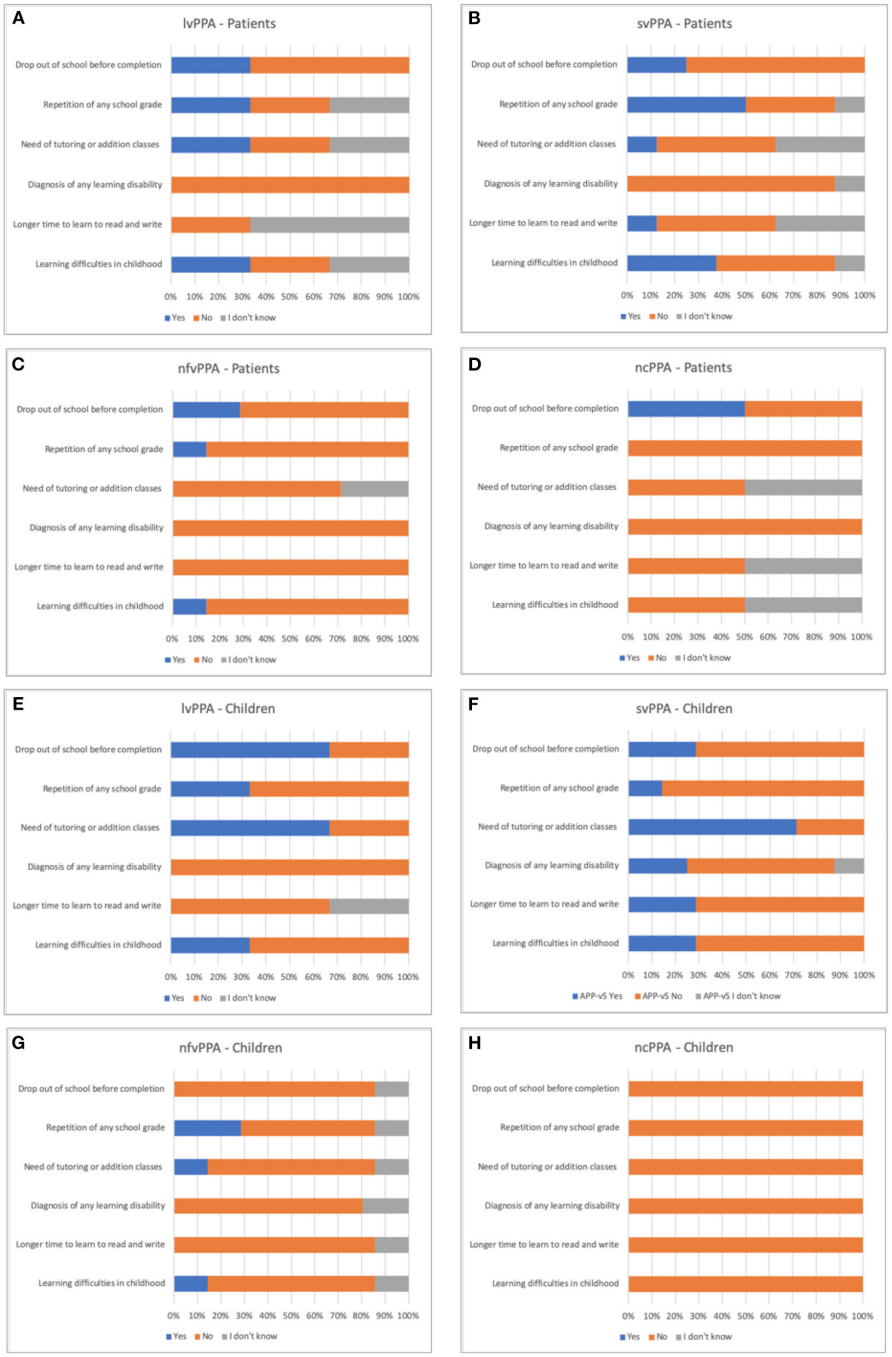
Description of the percentage of responses in the questionnaire of learning difficulties history of the PPA variants. AD, Alzheimer's disease; PPA, primary progressive aphasia; lvPPA, logopenic variant primary progressive aphasia; svPPA, semantic variant primary progressive aphasia; nfvPPA, nonfluent/agrammatic variant primary progressive aphasia; ncPPA, non-classifiable primary progressive aphasia. **(A)** responses of lvPPA patients; **(B)** responses of svPPA patients; **(C)** responses of nfvPPA patients; **(D)** responses of ncPPA patients; **(E)** responses of lvPPA children; **(F)** responses of svPPA children; **(G)** responses of nfvPPA children; **(H)** responses of ncPPA children.

Statistical analyses were conducted, comparing the responses between PPA and AD groups, as shown in Figure 1, and among the PPA variants (Figure 2). Regarding the comparison between patients with PPA and AD, and their children, no significant differences were observed regarding the occurrence of: a report of learning difficulties in childhood (patients, *p* = 0.32; children, *p* = 0.85); take it longer than children the same age to learn to read and write (patients, *p* = 0.71; children, *p* = 0.54); history of diagnosis of any learning disability (patients, *p* = 0.36; children, *p* = 0.25); need for tutoring or addition classes due to learning difficulties (patients, *p* = 0.35; children, *p* = 0.20); school dropout (children, *p* = 0.09); repetition of any school grade (patients, *p* = 0.14). However, there was a significantly higher occurrence of school dropout among people with AD, when compared to people with PPA (*p* < 0.01), and a significantly higher occurrence of repetition among children of people with AD when compared to PPA (*p* < 0.01).

The same variables were compared among the PPA variants (svPPA, lvPPA, nfvPPA, and unclassified cases of PPA). There was no statistically significant difference for any of the variables studied for both people with PPA and their children: history of learning difficulties (patients, *p* = 0.41; children, *p* = 0.79); take it longer than children the same age to learn to read and write (patients, *p* = 0.278; children, *p* = 0.196); history of diagnosis of any learning disability (patients, *p* = 0.64; children, *p* = 0.56); need for tutoring or addition classes due to learning difficulties (patients, *p* = 0.75; children, *p* = 0.10); school dropout (patients, *p* = 0.92; children, *p* = 0.12); repetition of any school grade (patients, *p* = 0.30; children = 0.70).

## Discussion

Learning disabilities have been treated as a potential risk factor in the development of PPA. There is evidence indicating that the history of learning disabilities, such as dyslexia, is significantly higher in patients with PPA and their first-degree relatives than in healthy controls or in patients with the behavioral variant of FTD or with AD (7, 9, 11, 15–17).

One of the main studies that demonstrated the association between PPA and learning disabilities is the study by Rogalski et al. (11). The study evaluated 699 subjects splitted into three groups of dementia: PPA, typical AD and behavioral variant of FTD, and a control group of healthy elderly people. This research investigated the occurrence of learning difficulties in patients and their first-degree relatives through self-reported questions about school learning. The results indicated higher concentrations of reading and writing difficulties in the families of individuals with PPA. One hypothesis to explain this association is the existence of susceptibility genes that could interfere with the initial language development, leading to developmental dyslexia in some individuals, while, in other cases, the effect could remain compensated for years but reappears as PPA in adulthood due to selective vulnerability of the language cortex to neurodegeneration (7, 11, 15). It is believed that some genetic risk factors linked to dyslexia may interact with the neurodegenerative process and increase the impact on the language network (7).

The fact that some neuropathological entities can cause PPA in some individuals while causing other dementias (amnestic or behavioral) in others justifies the search for susceptibility factors that interact with the neurodegenerative disease to collaborate with the differential diagnosis (7). Due to the difficulties to differentiate syndromes, such as PPA, AD, and the behavioral variant of FTD, studies have sought to gain a better understanding of the pathological processes involved in each disease.

Based on the pieces of evidence, the present study aimed to verify whether this association is also present in a sample of Brazilian individuals with PPA who are native speakers of the Brazilian Portuguese. However, unlike the findings in the literature, we did not find in our sample a higher occurrence of learning disabilities or difficulties among people with PPA or even among their children when compared to the group with AD and their children. Instead, there was an association between AD and the occurrence of school dropout and grade repetition, respectively, in people with AD and in the children of people with AD.

The non-association between the aspects evaluated and PPA may have occurred for different reasons. PPA is a disease centered on language disorders, and, therefore, it is possible that some risk factors are dependent on the language spoken by the subject. Our results may indicate that the behavior and development of the disease may be different in speakers of other languages than English since most of the studies produced so far focused on native English speakers (7, 9, 11, 16, 17). Other possibility is that, at the time when the generation of people investigated in this study was in school, the knowledge of learning disabilities among education and health professionals was scarce, and such conditions were not investigated, much less diagnosed. And last but not least, the absence of significant associations between the aspects evaluated and PPA could be due to the small sample of our study since we can observe a trend for elevated prevalence of learning difficulties in childhood of people with lvPPA and svPPA and their children. This trend is in line with a previous study that found higher prevalence of history of learning disabilities among the lvPPA (9), which is most often caused by AD pathology. The authors of that study argue that this finding suggests a susceptibility of the neural networks involved in the most commonly described form of dyslexia and lvPPA since they share similar cognitive (phonological) and anatomical (posterior temporoparietal) substrates.

For this reason, our questionnaire aimed not only to know if the participants had been diagnosed with learning disabilities but also the occurrence of other factors that could be suggestive of these difficulties, such as grade repetition, school dropout, the need for tutoring, or addition classes due to learning difficulties and delay in learning to read and write. However, all of these factors are also dependent on socioeconomic aspects, such as social inequality and the difficulty in access to basic education, which are relevant characteristics of the Brazilian society (18). Therefore, this argument could also explain the higher incidence of school dropout and grade repetition, respectively, among people with AD and their children since, in our sample participants with AD (and probably their family members) had a lower educational level than people with PPA.

The findings of our study related to the AD sample could also be supported by the literature that describes a possible association between other disorders that interfere with learning, such as attention deficit hyperactivity disorder (ADHD). Previous ADHD was associated with a higher risk of developing dementias other than PPA, such as AD and dementia with Lewy bodies (DLB) (19). Golimstok et al. (20) in a case-control study identified that ADHD symptoms were present at a significantly higher rate in patients later diagnosed with DLB. In agreement with these findings, Tzeng et al. (21) showed that, in a health service in Taiwan, over a period of 10 years, adults with ADHD were 3.4 times more likely to be diagnosed with dementia when compared to controls matched by gender and age without ADHD. However, Ivanchak et al. (22) did not find definitive evidence of association between ADHD in childhood and late degeneration related to dementia. In this study, a scale was applied to identify suspected cases of ADHD in 310 geriatric individuals with normal cognition, mild cognitive impairment, and diagnosis of dementia, and no associations were found between the variables. Thus, the correlation between ADHD and AD remains in need of further investigation and more accurate data to be confirmed. Such investigations may help to elucidate findings such as those in our research.

The main limitations of this study are the small sample size, especially the sample size of the different variants of the PPA, and the sociodemographic differences between the PPA and AD groups. These limitations are consequences of the difficulties in the detection and diagnosis of individuals with PPA in the Brazilian context. Although it is considered a low-frequency syndrome, research and clinical knowledge about PPA are still scarce (13). The knowledge of the diagnostic criteria and the differential factors between PPA and AD in Brazil is discussed in the study of Beber and Chaves (23) that indicates a tendency of the clinicians to generalize memory complaints toward a single diagnosis, identifying almost all these patients with FTDs as AD or leaving them undiagnosed. In addition, when patients with PPA receive the diagnosis, many of them are already in advanced stages of the disease, which makes it difficult to include this population in clinical research. Other limitations were the absence of a healthy control group and the use of a non-validated questionnaire to investigate the history of learning disabilities and related aspects. Despite that, we still consider that it is more reliable to interview participants using a non-validated but structured questionnaire than collecting data from medical records since information from medical records might not be collected from a standardized way. Finally, our study obtained information from caregivers, and some of them might not be lived with the participants during their childhood. To minimize this bias, we asked for the caregiver that had the best knowledge about the patient.

Despite the challenges of doing research with individuals with PPA in Brazil, we present the first evidence about the relationship between learning disabilities/difficulties and PPA among native Brazilian Portuguese speakers. We consider this an inaugural study on this topic, and we call attention for the fact that the results should be analyzed carefully, considering the limitations of the study since they indicate the importance of further investigations with larger and more homogeneous samples, and with neuroimaging and neuropsychological data. Long-term monitoring of patient cohorts can also favor the better understanding of the relationship between PPA and learning disabilities.

## Data Availability Statement

The raw data supporting the conclusions of this article will be made available by the authors, without undue reservation.

## Ethics Statement

The studies involving human participants were reviewed and approved by UFCSPA. The patients/participants provided their written informed consent to participate in this study.

## Author Contributions

TR and BB contributed to conception and design of the study. TR worked on the data collection. TM, PC, FS, and LF contributed to the participant's selection and recruitment. BC was the coordinator of the study. All authors contributed to manuscript revision, read, and approved the submitted version.

## Funding

Coordenação de Aperfeiçoamento de Pessoal de Nível Superior (CAPES) funded the master's degree scholarship of TR. CAPES is an agency of the ministry of education of the Brazilian government and supports graduate students in Brazil.

## Conflict of Interest

The authors declare that the research was conducted in the absence of any commercial or financial relationships that could be construed as a potential conflict of interest.

## Publisher's Note

All claims expressed in this article are solely those of the authors and do not necessarily represent those of their affiliated organizations, or those of the publisher, the editors and the reviewers. Any product that may be evaluated in this article, or claim that may be made by its manufacturer, is not guaranteed or endorsed by the publisher.
