# Supplementary material for: Investigation of the Association Between History of Learning Disabilities and Primary Progressive Aphasia in Brazilian Portuguese Speakers
Source: Front Neurol. 2022 Feb 28;13:703729. doi: 10.3389/fneur.2022.703729 (PMC8918792; doi:10.3389/fneur.2022.703729)
Supplement: Supplementary file 1 [file Data_Sheet_1.pdf]

Appendix 1 - Questionnaire about history of learning disabilities and difficulties in people with primary progressive aphasia and Alzheimer’s disease

**Questionnaire about history of learning disabilities and difficulties in people with primary progressive aphasia and Alzheimer’s disease – English Version**
**1. Personal and sociodemographic data**<br>*(These data could be obtained from the medical records and then confirmed by the caregiver)*
1.1 Participant (patient)
Name:
Sex: ( ) Female ( ) Male
Age:
Years of Education:
Race: ( ) White ( ) Mixed ( )Black ( ) Indigenous ( ) Yellow
Hand-Dominance: **( )** Right-handed ( ) Left-handed
1.2 Caregiver
Name:
Sex: ( ) Female ( ) Male
Age:
Years of Education:
Race: ( ) White ( ) Mixed ( )Black ( ) Indigenous ( ) Yellow
Relationship with the patient:
**2. Clinical data of patient**<br>*(These data could be obtained from the medical records and then confirmed by the caregiver)*
Neurological Diagnosis:<br>( ) Non-fluent PPA ( ) Semantic PPA ( ) Logopenic PPA<br>( ) Non-classifiable PPA ( ) Alzheimer's disease
Age of first symptoms:
**3. History of learning disabilities or difficulties**
3.1 PPA/AD patients
Did you have any learning difficulties when you were a child?<br>( ) Yes ( ) No ( ) I don't know
Did it take you longer than children the same age as you to learn to read and write?<br>( ) Yes ( ) No ( ) I don't know
Did you have a diagnosis of any of these learning disabilities?<br>( ) Dyslexia ( ) Dyscalculia ( ) Dysortography ( ) Dysgraphia ( ) ADHD<br>( ) No ( ) I don't know
Did you need tutoring or addition classes due to learning difficulties?<br>( ) Yes ( ) No ( ) I don't know
Did you repeat/retent any school grade?<br>( ) Yes ( ) No ( ) I don't know
Did you drop out of school before completion?<br>( ) Yes ( ) No ( ) I don't know Why? ____________________________
3.2 Patient’s children
Did any of your children have learning difficulties when they were a child<br>( ) Yes ( ) No ( ) I don't know
Did any of your children take longer than children the same age as them to learn to read and write?<br>( ) Yes ( ) No ( ) I don't know
Have any of your children ever been diagnosed with any of these learning disorders?<br>( ) Dyslexia ( ) Dyscalculia ( ) Dysortography ( ) Dysgraphia ( ) ADHD<br>( ) No ( ) I don't know
Did any of your children need tutoring or addition classes due to learning difficulties?<br>( ) Yes ( ) No ( ) I don't know
Did any of your children repeat/retent any school grade?<br>( ) Yes ( ) No ( ) I don't know Why? ____________________________
Did any of your childern drop out of school before completion?<br>( ) Yes ( ) No ( ) I don't know Why? ____________________________

**Questionário sobre história de transtornos e dificuldades de aprendizagem em pessoas com afasia progressiva primária e doença de Alzheimer – Versão em Português**
**1. Dados pessoais e sociodemográgicos**<br>*(Estes dados podem ser obtidos dos prontuários médicos e depois confirmados com o cuidador)*
1.1 Participante (paciente)
Nome:
Sexo: ( ) Feminino ( ) Masculino
Idade:
Anos de escolaridade:
Raça: ( ) Branco ( ) Pardo ( )Preto ( ) Indígena ( ) Amarelo
Dominância Manual: ( ) Destro ( ) Canhoto
1.2 Cuidador
Nome:
Sexo: ( ) Feminino ( ) Masculino
Idade:
Anos de escolaridade:
Raça: ( ) Branco ( ) Pardo ( )Preto ( ) Indígena ( ) Amarelo
Relação com o paciente:
**2. Dados clínicos do paciente**<br>*(Estes dados podem ser obtidos dos prontuários médicos e depois confirmados com o cuidador)*
Diagnóstico neurológico:<br>( ) APP não-fluente ( ) APP semântica ( ) APP logopênica<br>( ) APP não-classificável ( ) Doença de Alzheimer
Idade dos primeiros sintomas:
**3. História de transtornos ou dificuldades de aprendizagem**
3.1 Paciente com APP/DA
Você apresentou dificuldades escolares durante a infância?<br>( ) Sim ( ) Não ( ) Não sei
Você levou mais tempo que as crianças da mesma idade que você para aprender a ler e escrever?<br>( ) Sim ( ) Não ( ) Não sei
Você já foi diagnosticado com algum destes transtornos de aprendizagem?<br>( ) Dislexia ( ) Discalculia ( ) Disortografia ( ) Disgrafia ( ) TDAH<br>( ) Não ( ) Não sei
Você necessitou de reforço escolar devido às suas dificuldades de aprendizagem?<br>( ) Sim ( ) Não ( ) Não sei
Você repetiu de ano durante a fase escolar?<br>( ) Sim ( ) Não ( ) Não sei
Você abandonou os estudos antes do término?<br>( ) Sim ( ) Não ( ) Não sei Por quê? ___________________________
3.2 Filhos dos pacientes
Algum de seus filhos teve dificuldades de aprendizagem durante a infância?<br>( ) Sim ( ) Não ( ) Não sei
Algum de seus filhos levou mais tempo que as crianças da mesma idade para aprender a ler e escrever?<br>( ) Sim ( ) Não ( ) Não sei
Algum de seus filhos já foi diagnosticado com algum destes transtornos de aprendizagem?<br>( ) Dislexia ( ) Discalculia ( ) Disortografia ( ) Disgrafia ( ) TDAH<br>( ) Não ( ) Não sei
Algum de seus filhos necessitou de reforço escolar devido às dificuldades de aprendizagem?<br>( ) Sim ( ) Não ( ) Não sei
Algum de seus filhos repetiu de ano durante a fase escolar?<br>( ) Sim ( ) Não ( ) Não sei
Algum de seus filhos abandonou os estudos antes do término?<br>( ) Sim ( ) Não ( ) Não sei Por quê? ___________________________
